# Molecular Mechanism of HSF1-Upregulated ALDH2 by PKC in Ameliorating Pressure Overload-Induced Heart Failure in Mice

**DOI:** 10.1155/2020/3481623

**Published:** 2020-06-13

**Authors:** Endong Ji, Tiantian Jiao, Yunli Shen, Yunjia Xu, Yuanqing Sun, Zichun Cai, Qi Zhang, Jiming Li

**Affiliations:** ^1^Department of Cardiology, East Hospital, Tongji University School of Medicine, Shanghai 200120, China; ^2^Shanghai East Hospital of Clinical Medical College, Nanjing Medical University, Nanjing 211166, China

## Abstract

Evidences abound that HSF1 and ALDH2 are of cardioprotective effect, yet there is still no report on whether HSF1 can regulate ALDH2 to delay the occurrence of heart failure. We first established the pressure overload-induced heart failure model of mice by transverse aortic constriction (TAC) and discovered that, in the forming period of heart failure, changes of HSF1 and ALDH2 expression recorded the consistent trend. When HSF1 was upregulated/downregulated to delay/promote the occurrence of heart failure, PKC and ALDH2 also showed increased/decreased expression. And when ALDH2 was upregulated/downregulated, the role of HSF1 in delaying the occurrence of heart failure strengthened/weakened. Next, we used mechanical stretch to establish a pressure-stimulated myocardial hypertrophy model and discovered an increased expression of both HSF1 and ALDH2. When HSF1 was upregulated/downregulated to increase/decrease the expression of myocardial hypertrophy gene beta-MHC, PKC and ALDH2 recorded an increased/decreased expression. When an inhibitor was used to downregulate the expression of PKC in cardiomyocytes, we found that the role of HSF1 in upregulating ALDH2 beta-MHC weakened. These findings suggest that HSF1 can upregulate the expression of ALDH2 via PKC to promote pressure-stimulated myocardial compensatory hypertrophy, which is an important molecular pathway for HSF1 to ameliorate heart failure.

## 1. Introduction

As a common cardiovascular disease, heart failure is the unavoidable outcome of most heart diseases, and the most important cause lies with injury to the myocardium [1]. Though there are lots of medications for myocardial injury, including the angiotensin II-converting enzyme inhibitor and AT1 receptor blocker, yet treating myocardial injury is still not satisfactory. When cardiomyocytes have adverse stimulations, the outcomes whether they can survive or die depend on the endogenous protective mechanism. How to activate the mechanism in the early stage of myocardial injury becomes a key issue for protecting cardiomyocytes and preventing heart failure.

HSF1 and ALDH2 are potential endogenous cardioprotective factors, and there are already lots of evidences for their cardioprotective effect [2], but it is not yet reported whether HSF1 can regulate ALDH2 to delay the occurrence of heart failure. Exploring the mechanism can enrich the theory of endogenous protection in response to myocardial injury. Research on endogenous factors HSF1 and ALDH2 and development of medications that can enhance their activity or expression will hopefully lead to solutions for preventing or improving myocardial injury by protecting cardiomyocytes, inhibiting apoptosis, and promoting angiogenesis, which is of significant importance for effective prevention and treatment of heart failure. Through animal and cell experiments, we demonstrated the effect of HSF1 in promoting pressure-stimulated myocardial hypertrophy and further ameliorating heart failure by upregulating ALDH2 via PKC, providing a novel theoretical basis for exploring treatment approaches of heart failure.

## 2. Materials and Methods

### 2.1. Animals and Treatment

Adult male WT mice (C57BL/6, 8 weeks old) were obtained from the Shanghai Animal Administration Center (Shanghai, China). HSF1 transgene (TG) and HSF1 knockout (KO, HSF1^+/-^) mice were generated as previously described [3]. To generate a pressure overload-induced hypertrophy and heart failure model, the transverse aortic constriction (TAC) model was performed on animals which were randomly assigned to the following groups (*n* = 5 each): (1) sham group (sham-operated mice), (2) WT+TAC group (wild-type mice+TAC), (3) HSF1 TG+TAC group (HSF1 transgene mice+TAC), and (4) HSF1 KO+TAC group (HSF1 knockout mice+ TAC), and observed at day 3, day 7, day 14, and day 28 postsurgery. In brief, mice were anesthetized with isoflurane and placed in a supine position; the chest was opened and the transverse aortic constriction was dissected free of the surrounding tissues and muscles at the aortic arch level. A 6-0 nylon suture was tied around the aorta with a blunt 27-gauge needle which was removed after the ligation.

To investigate the role of HSF1 and ALDH2 in the protective effects of HSF1 transgene mouse cardiac remodeling and heart function, HSF1 transgene (TG) mice with TAC were divided into the following groups (*n* = 5 each): (1) Ad-ALDH2 group, (2) Ad-control group, and (3) Ad-ALDH2-shRNA group. After HSF1 transgene mice treated with TAC for 2 weeks, adenovirus-expressing ALDH2 (Ad-ALDH2) and Ad-expressing a short hairpin (sh) RNA targeted to ALDH2 (Ad-ALDH2-shRNA) purchased from Hanbio Technology Ltd. (Shanghai, China) were applied to transfect mice through intramyocardial injection (1 × 10^10^ viral particles (vp) per mouse) [4]. Four weeks after TAC, echocardiography measurements were done and then mouse heart tissues were collected.

The animal study was approved by the Animal Care Committee and Animal Ethics Committee at the Tongji University. All work performed under animal protocols approved by the Institutional Animal Care and Use Committee and conformed to the ‘Guide for the Care and Use of Laboratory Animals' (NIH Publication 85–23, revised 1996).

### 2.2. Cell Culture and Transfection

Cardiomyocytes (CM) were isolated from the ventricles of neonatal C57BL/6 mice (1 to 3 days old). The dissociated cells were cultured in Dulbecco's Modified Eagle's Medium (DMEM) supplemented with 10% fetal bovine serum (Gibco) and penicillin (100 U/ml)/streptomycin (100 *μ*g/ml) following preplating for 2 hours to exclude cells other than cardiomyocytes in a 5% CO2 incubator at 37°C. Culture medium was renewed at 2–3 days interval. Cells were stretched with silica gel distractor as mechanical stretch (MS) treatment.

We knocked down or overexpressed HSF1 by using adenoviruses harboring HSF1 (Ad-HSF1) and HSF1 shRNA (Ad-HSF1-shRNA) purchased from Hanbio Technology Ltd. (Shanghai, China) according to the manufacturer's instructions. The titers of adenoviruses used in this study were 1 × 10^10^ PFU/ml, and the multiplicity of infection used was 80 : 1. Cardiomyocytes were divided into different groups in accordance with different transfection sequences: (1) control: cardiomyocytes without mechanical stretch; (2) MS: cardiomyocytes with mechanical stretch 48 h; (3) Ad-HSF1-shRNA+MS: cardiomyocytes infected by adenovirus-expressing HSF1 shRNA with mechanical stretch 48 h; (4) Ad-HSF1+MS: cardiomyocytes infected by adenovirus-expressing HSF1 with mechanical stretch 48 h; and (5) Ad-HSF1+MS+PKC inhibitor: cardiomyocytes infected by adenovirus-expressing HSF1 and treated with protein kinase C (PKC) inhibitor sotrastaurin (Selleck Chemicals,Houston, TX) 1 *μ*M for 1 hour [5] and mechanical stretch 48 h.

### 2.3. Coimmunoprecipitation Assay

To determine the binding of HSF1/HSP70 and ALDH2 in vitro, transfected cells were lysed with a buffer (150 mmol/l NaCl, 1 mmol/l EDTA, 50 mmol/l Tris-HCl, 5% glycerol, 0.1% NP-40, and cocktails) at 4°C for 30 min. Then, an antibody was added to the mixture for 10 min followed by addition of protein A/G-agarose beads for 30 min. The concentration of the protein was determined by the BCA assay. Co-IP was performed according to a previous study [6]. Bound proteins were analyzed by Western blotting using corresponding antibodies.

### 2.4. Luciferase Reporter Gene Assay

For the reporter assay, cardiomyocytes were cultured into confocal dishes and performed different cotransfections of PBIND vector, PACT-MyoD control vector, pG5luc vector, pBIND-ALDH2, pACT-HSF1, and pACT-HSPA1A. RLU (relative light unit) was measured by the Modulus determinator (Turner Biosystems, USA).

### 2.5. Echocardiography

Transthoracic echocardiography was performed to examine cardiac morphology and cardiac function by using an animal-specific instrument (Visual Sonics Vevo770, VisualSonics Inc., Toronto, Canada). Mice were anesthetized and M-mode images were recorded. All data were averaged measurements of at least 5 cardiac cycles of every mouse in M-mode.

### 2.6. Invasive Hemodynamic Study

Carotid artery pressure was evaluated 4 weeks later after surgery [7]. In brief, a micromanometer (Millar 1.4F, SPR 835; Millar Instruments, Houston, TX) which was connected to a Power Laboratory system (AD Instruments, Castle Hill, New South Wales, Australia) was inserted through the right common carotid artery to obtain the carotid artery pressure.

### 2.7. Histological Analysis

Mice were euthanized, and then, each heart was excised, rinsed in normal saline, and fixed in 4% neutral formaldehyde at room temperature for more than 24 h for histological analysis. The specimens were embedded in paraffin and 4 *μ*m thick serial sections were prepared in the short axis at the papillary muscle lever and stained with hematoxylin and eosin (HE) and Masson trichrome. For measurement, five random fields from each section were chosen and 5 sections from each heart were examined.

### 2.8. Reverse Transcription Polymerase Chain Reaction (RT-PCR) Analysis

Total RNA was extracted from heart tissues or cells using the TRIzol reagent (Invitrogen) according to the manufacturer's instructions, purified with RNeasy Mini Kit (QIAGEN, Valencia, CA), and treated with RNase-free DNase (QIAGEN, CA) to eliminate genomic DNA contamination. Quantitative reverse transcriptase PCR (qRT-PCR) was conducted under the following conditions: 30 s at 95°C, 40 cycles of 5 s at 95°C, 34 s at 60°C, and 30 s at 72°C. RNA equivalents were normalized to simultaneously determine glyceraldehyde-3-phosphate dehydrogenase (GAPDH) mRNA levels in each sample. All PCRs were performed in triplicate. The difference in gene expression was evaluated using the threshold cycle difference between relevant genes and the internal controls using the method of relative gene expression 2^–*ΔΔ*CT^. For the mouse gene expression, specific primer pairs used in the PCR were as follows:

1. HSF1 sense (5′-TCTCCTGTCCTGTGTGCCTAGC-3′) and HSF1 antisense (5′-CAGGTCAACTGCCTACACAGACC-3′)
2. ALDH2 sense (5′--CCTGAGCCGAATGCTTTTAAG-3′) and ALDH2 antisense (5′-CTCACGCTCCTTACTGGAC-3′)
3. Beta-MHC sense (5′- CAGCAGTTCTTCAACCACCA-3′) and beta-MHC antisense (5′- TCTCGATGAGGTCAATGCAG-3′)
4. PKC sense (5′- ATTGCTGCTTCCAGACCAAG-3′) and PKC antisense (5′- GGCATAGAACCGAGAACGAG-3′)
5. GAPDH sense (5′-GGAAAGCTGTGGCGTGATGG-3′) and GAPDH antisense (5′-GTAGGCCATGAGGTCCACCA-3′).

### 2.9. Western Blotting

Methodologies for the protein extraction of heart tissues or cells were as described previously [8]. For Western blot, equal quantities of proteins (20-50 *μ*g/lane) were subjected to 8-12% SDS-PAGE, depending on the target proteins; electrotransferred onto polyvinylidene difluoride (PVDF) membranes; incubated with primary antibodies including polyclonal rabbit anti-mice HSF1 antibody, ALDH2 antibody, HSP90 antibody, beta-MHC antibody, PKC antibody, and GAPDH antibody (all purchased from Abcam Biotechnology, USA, 1 : 1000 dilution), and horseradish peroxidase-conjugated secondary antibody (Beyotime Biotech, China) was assessed on standard Western blotting. GAPDH was serving as the loading control for the respective protein analysis.

### 2.10. Statistical Analysis

All data are shown as mean ± standard deviation (SD). Multigroup comparisons were performed by one-way analysis of variance (ANOVA) followed by Tukey's test for post hoc analysis. Statistical analyses were performed with GraphPad Prism 7.0 software (GraphPad, San Diego, CA) and SPSS 20.0 software. A value of *p* < 0.05 was considered statistically significant difference.

## 3. Results

### 3.1. Correlation between HSF1 and ALDH2 in Mouse Heart Failure Model In Vivo

**Figure 1:**
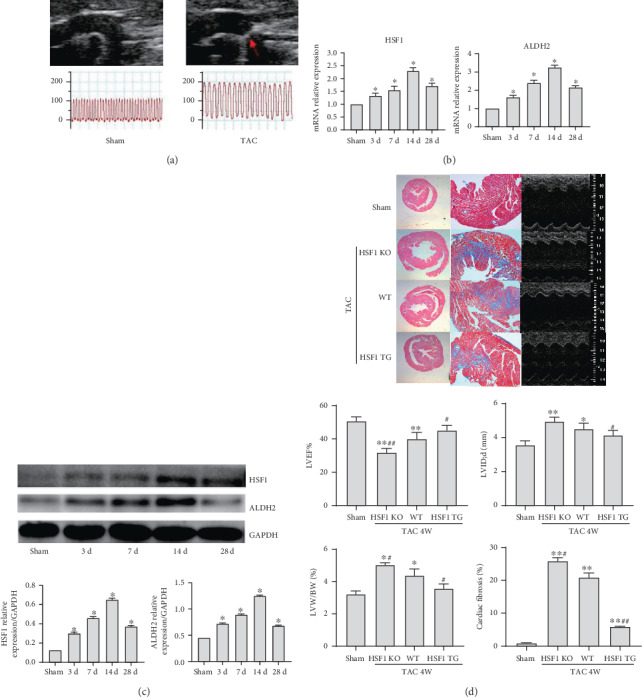
TAC pressure overload model, the expression of HSF1, ALDH2, and mouse cardiac remodeling and heart function after TAC 4 weeks. (a) Mouse TAC pressure overload model: mouse aortic constriction (the red arrow shows mouse aortic constriction ring measured by echocardiography) and common carotid artery pressure (measured by Millar micromanometer). (b) The relative mRNA level of the mouse heart tissue (sham and TAC wild-type mice). (c) The relative protein level of the mouse heart tissue (sham and TAC wild-type mice). (d) Mouse heart histologic section: HE (1.25x) and Masson (5.0x) staining and echocardiography measure (sham: sham-operated mice; WT: wild-type mice; HSF1 TG: HSF1 transgene mice; HSF1 KO: HSF1 knockout mice). LVW/BW: left ventricular weight/body weight; LVID, d: left ventricular internal dimension, diastolic; LVEF: left ventricular ejection fraction. ^∗^*p* < 0.05, ^∗∗^*p* < 0.005 vs. sham, ^#^*p* < 0.05, ^##^*p* < 0.005 vs. WT TAC, *n* = 5.

We establish the mouse pressure overload heart model by TAC (Figure 1(a)); animal mortality is less than 20%. We discovered that, in the forming period of heart failure, HSF1 and ALDH2 recorded the consistent trend in terms of expression of their mRNA and protein (Figures 1(b) and 1(c)), indicating that there exists a correlation between HSF1 and ALDH2. We further used HSF1 transgene and knockout mice after TAC for 4 weeks and observed that HSF1 transgene mice recorded improved heart failure (Figure 1(d)), which indicates that HSF1 probably plays its role in delaying the occurrence of heart failure via regulating ALDH2.

### 3.2. Role of ALDH2 in HSF1 Ameliorating Heart Failure In Vivo

**Figure 2:**
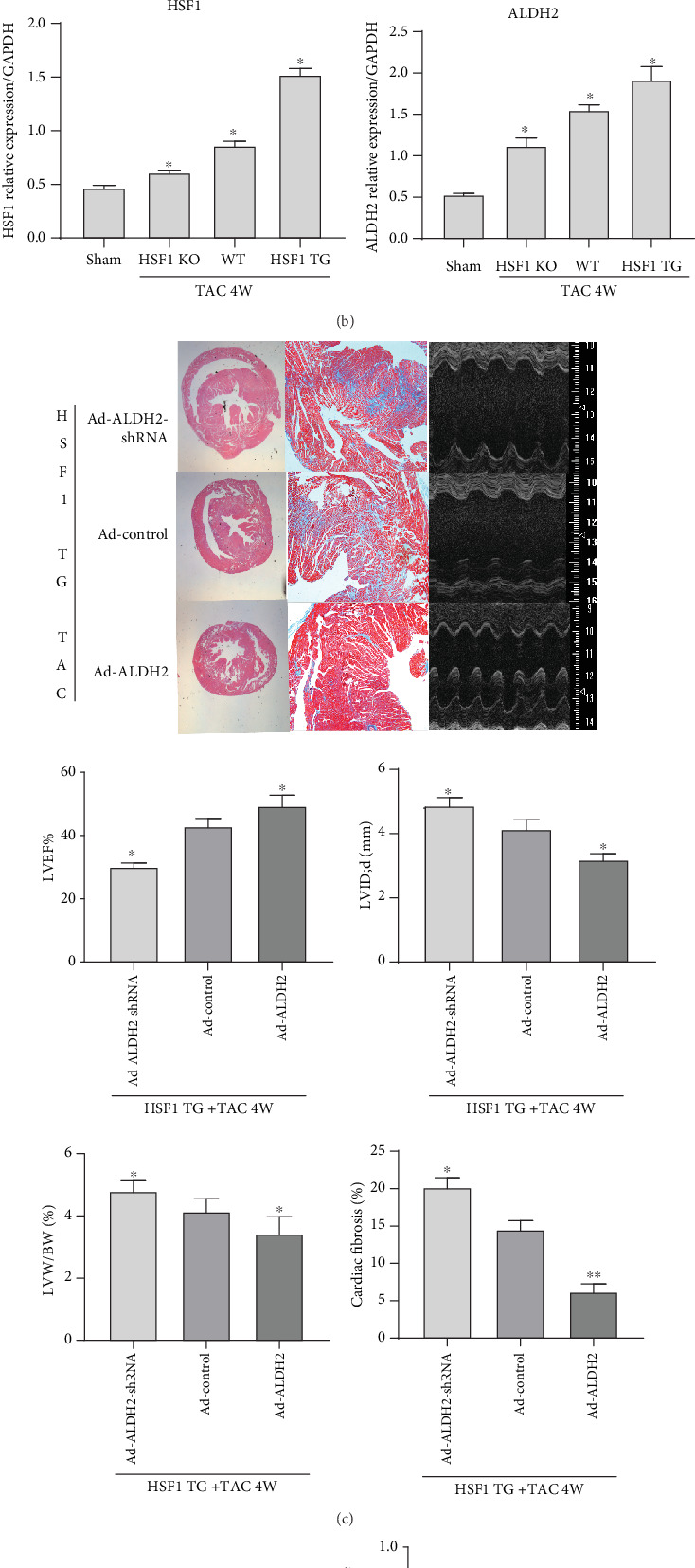
The expression of HSF1, ALDH2, and ALDH2 upregulated or downregulated HSF1 transgene mice cardiac remodeling and heart function after TAC 4 weeks. (a) The relative mRNA level of the mouse heart tissue (sham: sham-operated mice; WT: wild-type mice; HSF1 TG: HSF1 transgene mice; HSF1 KO: HSF1 knockout mice). (b) The relative mRNA and protein levels of the mouse heart tissue (sham: sham-operated mice; WT: wild-type mice; HSF1 TG: HSF1 transgene mice; HSF1 KO: HSF1 knockout mice). (c) TAC HSF1 transgene mice heart histologic section: HE (1.25x) and Masson (5.0x) staining and echocardiography measure. (d) The relative protein expression of ALDH2 in the mouse heart tissue in each group. Ad-ALDH2-shRNA: mice are injected with adenovirus-expressing ALDH2 shRNA; Ad-control: mice are injected with blank adenovirus; Ad-ALDH2: mice are injected with adenovirus-expressing ALDH2; LVW/BW: left ventricular weight/body weight; LVID, d: left ventricular internal dimension, diastolic; LVEF: left ventricular ejection fraction. ^∗^*p* < 0.05, ^∗∗^*p* < 0.005 vs. sham, ^#^*p* < 0.05, ^##^*p* < 0.005 vs. WT TAC, *n* = 5.

ALDH2 showed an increased expression of mRNA and protein in HSF1 transgene mice after TAC for 4 weeks, but HSF1 knockout mice presented the contrary results (Figures 2(a) and 2(b)), The above results showed that HSF1 can delay the occurrence of heart failure. To examine the role of ALDH2 in the cardioprotective effect of HSF1 in attenuating heart failure, we used the adenovirus transfection method to establish the ALDH2 upregulation/downregulation model of HSF1 transgene mice. Four weeks after TAC, when the expression of ALDH2 protein was downregulated, the effect of HSF1 in delaying heart failure weakened, and when ALDH2 was upregulated, the record was the contrary (Figures 2(c) and 2(d)). These results suggest that ALDH2 is a molecular pathway for the protective effect of HSF1 in attenuating heart failure.

### 3.3. Correlation between HSF2 and ALDH2 in Cardiomyocytes In Vitro

**Figure 3:**
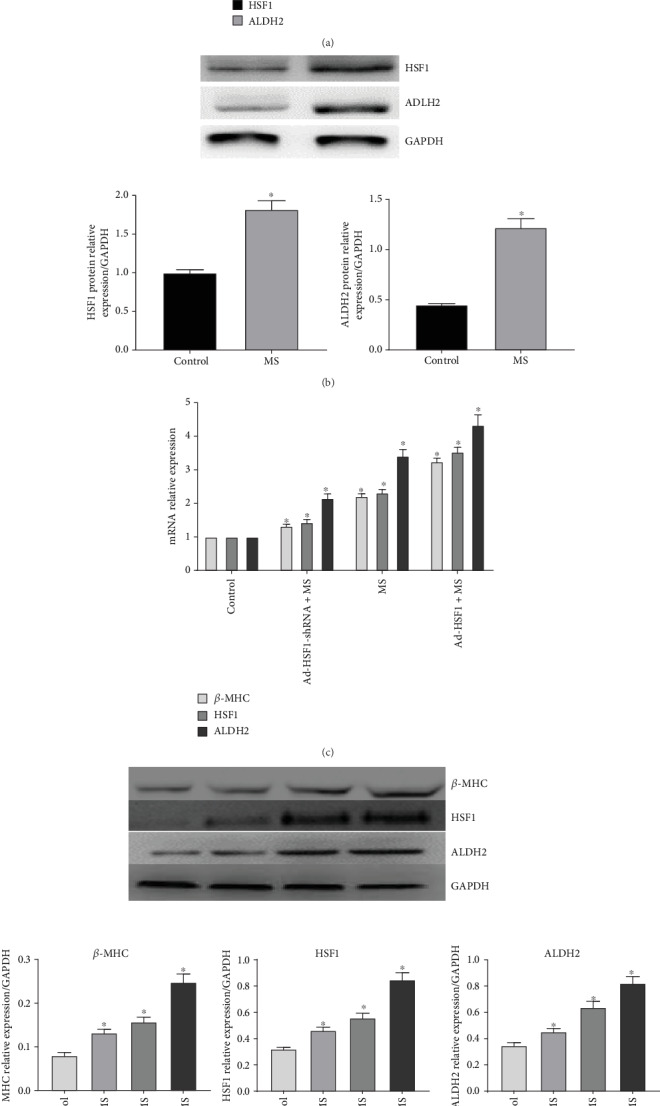
The relative HSF1, ALDH2, and beta-MHC expression levels of neonatal mouse cardiomyocytes after mechanical stretch in vitro (a, b) The relative HSF1, ALDH2 mRNA, and protein levels of neonatal mouse cardiomyocytes (control: cardiomyocytes without mechanical stretch; MS: cardiomyocytes with mechanical stretch 48 h) (c, d) The relative HSF1, ALDH2, and beta-MHC mRNA and protein levels of neonatal mouse cardiomyocytes (control: cardiomyocytes without mechanical stretch; MS: cardiomyocytes with mechanical stretch 48 h; Ad-HSF1-shRNA+MS: cardiomyocytes infected by adenovirus-expressing HSF1 shRNA with mechanical stretch 48 h; Ad-HSF1+MS: cardiomyocytes infected by adenovirus-expressing HSF1 with mechanical stretch 48 h). ). ^∗^*p* < 0.05, *n* = 5.

We used mechanical stretch to establish the myocardial hypertrophy model and observed that HSF1/HSP70 and ALDH2 in the cardiomyocytes both recorded increased expressions (Figures 3(a) and 3(b)), indicating that there exists a correlation between HSF1 and ALDH2. We further used adenovirus transfection to regulate the expression of HSF1 and observed that, when HSF1 upregulated the expression of hypertrophy gene beta-MHC, ALDH2 also presented an increased expression, and when HSF1 was downregulated, the result was the contrary (Figures 3(c) and 3(d)), indicating that HSF1 probably plays its cardioprotective role via regulating ALDH2.

### 3.4. Mechanism of Regulating ALDH2 by HSF1

To explore the pathway for HSF1 to regulate ALDH2, we used the heart organ of 4-week TAC mice and 48-hour stretched cardiomyocytes and applied coimmunoprecipitation for observation, but there was no positive result for the binding between HSF1 and ALDH2 (Figure S1). To further verify this result, we used the method of luciferase reporter gene, and no binding results between HSF1 and ALDH2 were found (Figure S2). Then, what is the mediator for HSF1 to play its role in regulating ALDH2? Previous studies on ischemia/reperfusion injury by American researchers showed that mitochondrial import of PKC*ε* is mediated by HSP90 and is required for cardiac protection against IR. Therefore, we speculate whether HSF1 promotes mitochondrial import of PKC*ε* by upregulating the expression of HSP90, thus promoting the expression of ALDH2.

**Figure 4:**
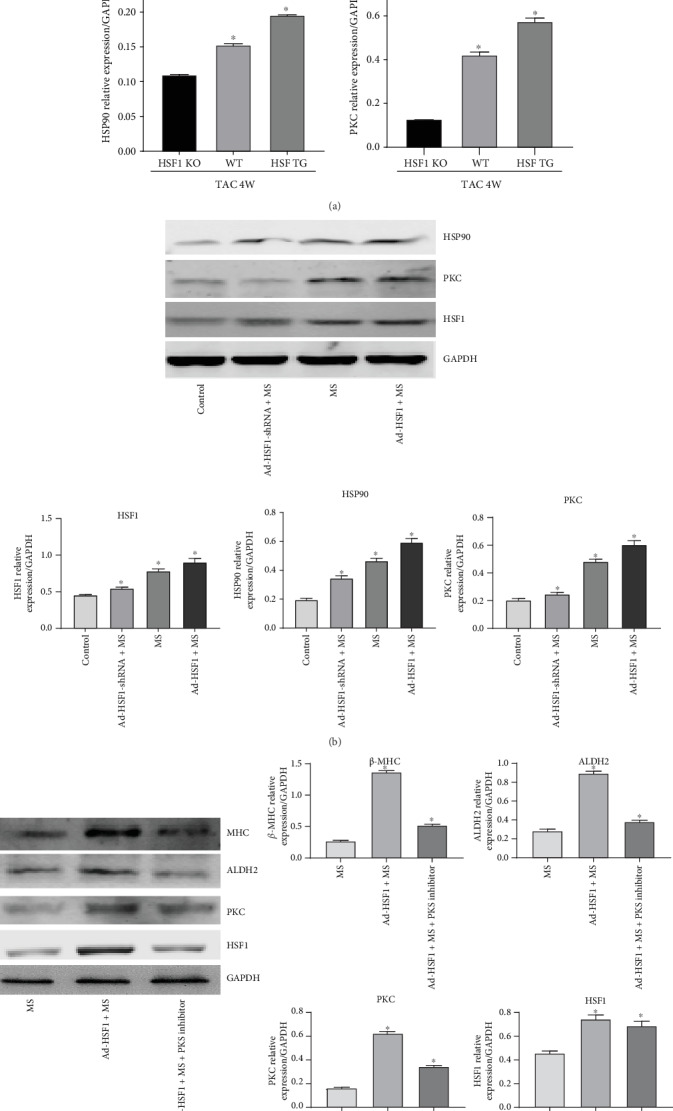
The expression of HSF1, PKC, ALDH2, HSP90, and beta-MHC in upregulated or downregulated HSF1 mouse heart tissue or cardiomyocytes. (a) The protein level of PKC and HSF90 in the WT, HSF1 TG, and HSF1 KO mouse heart tissues after TAC for 4 weeks. (sham: sham-operated mice; WT: wild-type mice; HSF1 TG: HSF1 transgene mice; HSF1 KO: HSF1 knockout mice). (b) The protein level of HSF1, HSP90, and PKC in upregulated or downregulated HSF1 cardiomyocytes. (control: cardiomyocytes without mechanical stretch; MS: cardiomyocytes with mechanical stretch 48 h; Ad-HSF1-shRNA+MS: cardiomyocytes infected by adenovirus-expressing HSF1 shRNA with mechanical stretch 48 h; Ad-HSF1+MS: cardiomyocytes infected by adenovirus-expressing HSF1 with mechanical stretch 48 h). (c) The expression of HSF1, PKC, ALDH2, and beta-MHC in upregulated HSF1 cardiomyocytes (MS: cardiomyocytes with mechanical stretch 48 h; Ad-HSF1+MS: cardiomyocytes infected by adenovirus-expressing HSF1 with mechanical stretch 48 h; Ad-HSF1+MS+PKC inhibitor: cardiomyocytes infected by adenovirus-expressing HSF1and treated with PKC inhibitor with mechanical stretch 48 h). ∗*p* < 0.05, *n* = 5.

Next, in the TAC 4-week heart failure model in vivo, we also observed that when HSF1 was upregulated, HSP90 and PKC recorded an increased expression, and when HSF1 was downregulated, the result was the contrary (Figure 4(a)). In the hypertrophic cardiomyocytes in vitro, when HSF1 was upregulated, HSP90 and PKC recorded an increased expression, and when HSF1 was downregulated, the result was the contrary (Figure 4(b)). These results demonstrated that, in the heart organ and hypertrophic cardiomyocytes, HSF1 can regulate the expression of PKC by upregulating the expression of HSP90, and it was previously reported that PKC regulates the expression of ALDH2, suggesting that PKC is probably an important molecular pathway for HSF1 to regulate ALDH2. To examine the role of PKC in the regulation of ALDH2 by HSF1, we used adenovirus transfection to establish the cardiomyocyte model of HSF1 upregulation and after 48-hour mechanical stretch, observed that, when the inhibitor downregulated the expression of PKC, ALDH2 recorded a decreased expression and the expression of hypertrophy gene beta-MHC was reduced (Figure 4(c)). These results demonstrate that HSF1 changes the expression of ALDH2 by regulating PKC, which is a functioning pathway for HSF1 to protect hypertrophic cardiomyocytes.

## 4. Discussion

In recent years, the role of myocardial mitochondrial dysfunction in myocardial injury and heart failure gradually attracted attention [9, 10], but still little is known about its mechanism. Myocardial apoptosis is a major mechanism for heart failure [11], and the mitochondria play a very important role in the process of myocardial apoptosis [12]. As a kind of important aldehyde oxidase within the mitochondria, ALDH2 has the key function of oxidizing the intermediary metabolite acetaldehyde of ethyl alcohol into acetic acid. Research on the ALDH2 function in the past mainly focused on the metabolism of ethyl alcohol within the liver. Till 2003, there came a report that ALDH2 can play a protective role for neurocyte injury resulting from nonethyl alcohol [13]. Later, reports gradually appeared that ALDH2 is of protective effect for human umbilical vein endothelial cells and pulmonary epithelial cells [14, 15]. Regarding heart diseases, recent studies mainly focused on its protective effect for the heart. In 2008, *Science* reported that ALDH2 can delay heart failure after myocardial infarction [16]. Later, reports gradually came that ALDH2 is of protective effect for alcoholic heart injury, myocardial ischemia, and coronary vessels [17–19], identifying the protective role of ALDH2 against heart diseases. In 2009, *Trends Cardiovascular Medicine* predicted that ALDH2 would hopefully become a novel target for treatment of heart diseases [20].

Previous research demonstrated that both HSF1 and ALDH2 are critical endogenous protective factors of the heart and play an important protective role in heart diseases [21–23]. ALDH2 can ameliorate myocardial apoptosis from heart failure and delay the occurrence and development of heart failure [24, 25]. Studies on ischemia/reperfusion injury by Japanese researchers showed that HSF1 can activate PKC [26], and research on vascular endothelial cell also found that HSF1 can regulate PKC [27]. American researchers studied the relationship between the PKC*ε*/MAPK signaling pathway and ALDH2 within cardiomyocytes [28], identifying that ALDH2 was the downstream regulated substrate of PKC*ε*. These studies indicate that HSF1 probably regulates ALDH2 via PKC, yet it is still unknown whether HSF1 and ALDH2 can mutually regulate each other to mediate the endogenous protective mechanism of the heart.

Our research discovered that HSF1 is one of the upstream mediators of ALDH2. First, we used TAC to establish the pressure overload-induced heart failure model of mice and found that compared with the wild group, HSF1 transgene mice and knockout mice recorded no difference in ALDH2 expression before TAC. But when heart failure occurred four weeks after TAC, HSF1 transgene mice recorded better heart function, and at the same time, the expression of ALDH2 mRNA and protein recorded increases. HSF1 knockout mice presented worse heart function, and at the same time, the expression of ALDH2 mRNA and protein decreased. Additionally, when ALDH2 in the heart of the HSF1 transgene mice was downregulated, the role of HSF1 in delaying the occurrence of heart failure weakened, and when ALDH2 was upregulated, the result was the contrary, when the expression of HSF1 increased, that of PKC also increased. And when HSF1 was downregulated, PKC present corresponding changes. We also observed that, in the early stage of compensated heart hypertrophy, HSF1 and the protein recorded an increased expression. But as the pressure overload sustained, the expression of HSF1 mRNA and protein started to decrease when the heart was decompensated, and heart function also began to reduce. Additionally, ALDH2 recorded the consistent trend. Further in the research on cardiomyocytes in vitro, we found that, as the expression of myocardial hypertrophy gene increased, HSF1 and ALDH2 also correspondingly increased. We further used adenovirus to regulate HSF1 and found that, when HSF1 was upregulated, the expression of the myocardial hypertrophy gene also increased, and ALDH2 also recorded increases. When HSF1 was downregulated, the result was the contrary. When HSF1 played its protective role against myocardial hypertrophy injury resulting from mechanical stretch, the expression of PKC also recorded a similar changing trend. Additionally, we blocked PKC by using an inhibitor and observed that, when HSF1 was upregulated, the role of ALDH2 and the cardiac hypertrophy gene weakened. These results and previous studies suggest that, in the hypertrophic cardiomyocytes, PKC is a mediating factor for HSF1 to regulate ALDH2, and it is by regulating PKC at least partly that HSF1 upregulates the expression of ALDH2 and further plays its protective role in response to myocardial hypertrophy.

In summary, through animal and cell experiments, we explored a novel mechanism for the occurrence and development of heart failure and demonstrated that HSF1 is the upstream regulating factor for ALDH2, and one of the mediators is PKC. By upregulating expression of ALDH2 via mitochondrial import of PKC*ε* while upregulating HSP90, HSF1 can play the protective role in response to myocardial hypertrophy injury resulting from pressure stimulation and further delay the occurrence and development of heart failure of mice. This provides a novel theoretical basis for diagnosis and treatment of heart failure and enriches the theory in an endogenous protective mechanism against myocardial damages. Research on endogenous cardioprotective factors will hopefully lead to solutions for avoiding, preventing, or attenuating myocardial injury, offering a new target for exploring approaches for treatment of heart failure in the future.

## Data Availability

The data used to support the findings of this study are available from the corresponding author upon request.

## Supplementary Materials

Supplementary Materials Fig S1; Co-immunoprecipitation result for the binding between HSF1/HSP70 and ALDH2.Input lane was set as control to identify the target protein comprised in precleared protein lysis. IgG lane was set as negative control. Anti-ALDH2 lane was protein precipatated by ALDH2 hybridizing with HSF1 antibody. HSF1 was not detected. Anti-HSF1 lane was protein precipatated by HSF1 hybridizing with ALDH2 antibody. ALDH2 was not detected. These results of co-immunoprecipitation indicated no positive result for the binding between HSF1 and ALDH2. Fig S2; Luciferase reporter gene assay of ALDH2 interacts with HSF1 and HSPA1A.Data of RLU ratio on average calculated by the ratio of luciferase RLU and Renilla luciferase RLU present as a histogram. The positive and negative data were normal, the target gene data and the negative data were similar, indicated that there were no binding results between HSF1/HSPA1A and ALDH2.
