# Supplementary material for: Molecular Mechanism of HSF1-Upregulated ALDH2 by PKC in Ameliorating Pressure Overload-Induced Heart Failure in Mice
Source: Biomed Res Int. 2020 Jun 13;2020:3481623. doi: 10.1155/2020/3481623 (PMC7313111; doi:10.1155/2020/3481623)
Supplement: Supplementary Materials — Fig S1; Co-immunoprecipitation result for the binding between HSF1/HSP70 and ALDH2.Input lane was set as control to identify the target protein comprised in precleared protein lysis. IgG lane was set as negative control. Anti-ALDH2 lane was protein precipatated by ALDH2 hybridizing with HSF1 antibody. HSF1 was not detected. Anti-HSF1 lane was protein precipatated by HSF1 hybridizing with ALDH2 antibody. ALDH2 was not detected. These results of co-immunoprecipitation indicated no positive result for the binding between HSF1 and ALDH2. Fig S2; Luciferase reporter gene assay of ALDH2 interacts with HSF1 and HSPA1A.Data of RLU ratio on average calculated by the ratio of luciferase RLU and Renilla luciferase RLU present as a histogram. The positive and negative data were normal, the target gene data and the negative data were similar, indicated that there were no binding results between HSF1/HSPA1A and ALDH2. [file 3481623.f1.docx]

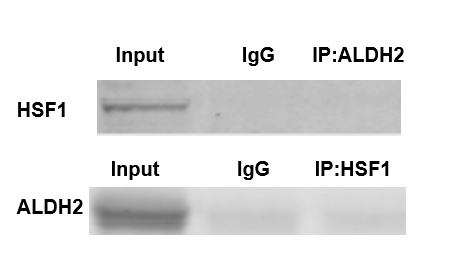


Fig S1; Co-immunoprecipitation result for the binding between HSF1/HSP70 and ALDH2.Input lane was set as control to identify the target protein comprised in precleared protein lysis. IgG lane was set as negative control. Anti-ALDH2 lane was protein precipatated by ALDH2 hybridizing with HSF1 antibody. HSF1 was not detected. Anti-HSF1 lane was protein precipatated by HSF1 hybridizing with ALDH2 antibody. ALDH2 was not detected. These results of co-immunoprecipitation indicated no positive result for the binding between HSF1 and ALDH2.


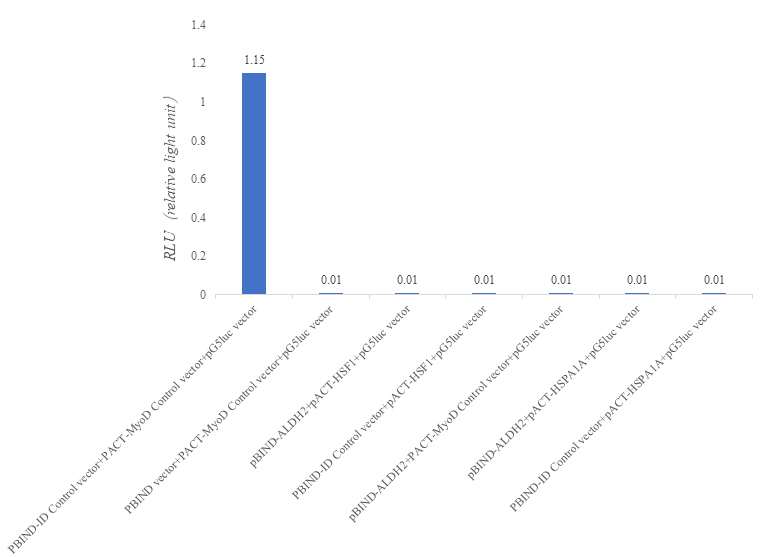


Fig S2; Luciferase reporter gene assay of ALDH2 interacts with HSF1 and HSPA1A.Data of RLU ratio on average calculated by the ratio of luciferase RLU and Renilla luciferase RLU present as a histogram. The positive and negative data were normal, the target gene data and the negative data were similar, indicated that there were no binding results between HSF1/HSPA1A and ALDH2.
